# Markers of Antioxidant Defense in Patients with Type 2 Diabetes

**DOI:** 10.1155/2016/2352361

**Published:** 2015-11-10

**Authors:** K. Gawlik, J. W. Naskalski, D. Fedak, D. Pawlica-Gosiewska, U. Grudzień, P. Dumnicka, M. T. Małecki, B. Solnica

**Affiliations:** ^1^Department of Diagnostics, Chair of Clinical Biochemistry, Jagiellonian University Medical College, 31-501 Krakow, Poland; ^2^Department of Medical Diagnostics, Jagiellonian University Medical College, 30-688 Krakow, Poland; ^3^Chair of Metabolic Diseases, Jagiellonian University Medical College, 31-501 Krakow, Poland

## Abstract

*Aims*. Diabetes is considered a state of increased oxidative stress. This study evaluates blood concentrations of selected markers of antioxidant defense in patients with type 2 diabetes. *Methods*. The study included 80 type 2 diabetes patients and 79 apparently healthy controls. Measured markers included ferric reducing ability of plasma (FRAP), reduced glutathione (GSH), glutathione peroxidase (GPx), glutathione reductase (GR), *γ*-glutamyltransferase (GGT) and uric acid serum, and plasma and/or hemolysate levels. *Results*. FRAP, uric acid, CRP, and GGT levels were significantly higher in patients with diabetes. Plasma and hemolysate GR was significantly higher whereas GPx activity was significantly lower in patients with diabetes. There were no significant differences in antioxidant defense markers between patients with and without chronic diabetes complications. Fasting serum glucose correlated with plasma GPx, plasma and hemolysate GR, FRAP, and serum GGT, and HbA1c correlated with serum GGT. Only FRAP and serum uric acid were significantly higher in obese (BMI > 30 kg/m^2^) patients with diabetes than in nonobese patients. *Conclusions*. Some components of antioxidant defense such as GR, uric acid, and GGT are increased in patients with type 2 diabetes. However, the whole system cannot compensate for an enhanced production of ROS as reflected by the trend toward decreased erythrocytes GSH.

## 1. Introduction

Persistent hyperglycemia secondary to insulin resistance and diminished insulin secretion in type 2 diabetes leads to progressing organ injuries known as late or chronic diabetes complications. It is believed that one of the underlying causes of microvascular and macrovascular diabetes complications is oxidative stress [1].

There are several suggested mechanisms linking hyperglycemia with increased production of reactive oxygen species (ROS). These mechanisms include increased mitochondrial synthesis of superoxide anion radical (O_2_^−^) [2], activation of the NF-*κ*B signaling pathway leading to inflammatory reaction and increased ROS production in phagocytes [3], increased glucose flux through the polyol pathway, and formation of the advanced glycation end products (AGE) enhancing oxidative stress [4].

In general, oxidative stress is caused by an imbalance between ROS generation and antioxidant defense mechanisms eliminating the superoxide anion radical and similar compounds. Antioxidant defense system consists of a series of specific enzymes, metal binding proteins, and a number of low molecular weight antioxidants such as ascorbate, cysteine, glutathione, and urate. Some elements of this system are measured in blood as markers of antioxidant defense. Diabetes is considered a state of increased oxidative stress but the published data referring to impairment of antioxidant defense in diabetic patients are contradictory [5, 6].

The aim of this study was to evaluate blood concentrations of selected markers of antioxidant defense in patients with type 2 diabetes and to assess the relationship between antioxidant defense and glycemic control and the presence of diabetes complications.

## 2. Materials and Methods

The study included 80 patients with type 2 diabetes—40 females and 40 males, aged from 30 to 80 years (mean 59.5 ± 11.5), hospitalized in the Department of Metabolic Diseases, University Hospital, Krakow, Poland. Mean disease duration from diagnosis was 8.5 years (4–12). In 77 (88.7%) patients arterial hypertension was diagnosed. Among studied patients 41 had chronic complications of diabetes including nephropathy in 15 patients, retinopathy in 21, neuropathy in 29, and diabetic foot in 6 patients. Exclusion criteria included the fifth stage of chronic kidney disease (GFR < 15 mL/min/1.73 m^2^), inflammatory diseases, cancer, and systemic diseases.

The control group was composed of 79 healthy subjects matched according to age (mean 55.2 ± 11; range 30–85 years) and sex with the studied patients (Table 1).

Healthy subjects were recruited from the individuals who went through regular medical check-up in the Department of Diagnostics, University Hospital, Krakow, Poland. They were selected among individuals with no history of cancer and systemic diseases, healthy at the time of study, and whose laboratory findings did not show inflammatory disease, prediabetes, or diabetes.

Participation required informed consent signed by all subjects enrolled both into the studied and into the control group. The study had the approval of Jagiellonian University Bioethics Commission number KBET/139/09/.

Blood samples for the study purposes were collected using S-Monovette-serum and S-Monovette-EDTA tubes (SARSTEDT AG&Co, Nümbrecht, Germany). Routine laboratory tests including fasting glucose, lipid profile, gamma-glutamyltransferase activity, and creatinine and uric acid concentrations were performed in serum. HbA1c levels were measured in whole blood.

The K_2_-EDTA blood samples were used for measurements of selected antioxidant defense markers in plasma (the ferric reducing ability of plasma (FRAP), glutathione peroxidase (GPx), and glutathione reductase (GR) activity) and in hemolysate (glutathione peroxidase (GPx) and glutathione reductase (GR) activity and reduced glutathione concentration (GSH)). These blood samples were centrifuged for 10 minutes at 3000 ×g and the obtained plasma was separated, aliquoted to microtubes, and stored frozen at −80°C from 1 to 12 months until testing. Remaining erythrocytes were separated from plasma, washed three times with isotonic saline, and hemolyzed by a fourfold dilution with deionized water. The obtained erythrocytes suspension was frozen and stored overnight at −80°C. Then hemolysate samples were thawed and centrifuged at 3000 ×g for 10 minutes to remove cell debris. The hemolysate samples were aliquoted to microtubes and stored frozen at −80°C for 1–12 months before use.

Hemoglobin concentration in hemolysate was measured using the cyanmethemoglobin method [7]. After mixing of hemolysate samples with diluted potassium ferricyanide and potassium cyanide solution at a slightly alkaline pH to form the stable cyanmethemoglobin, the absorbance was measured at 540 nm using microplate EL-800 reader (BioTek Instruments, Inc., USA). Hemolysate reduced glutathione concentration was determined using the method described by Beutler et al. employing 5,5′-dithiobis(nitrobezoic) acid forming with glutathione thiol groups colored adduct, with spectrophotometric measurement at 412 nm [8]. Glutathione peroxidase activity was measured in plasma and hemolysate based on the decrease of NADPH absorbance at 340 nm according to the method of Paglia and Valentine [9]. Similarly, glutathione reductase activity was measured in plasma and hemolysate using the method based on the reduction of glutathione (GSSG) in the presence of NADPH, which is oxidized to NADP^+^ [10]. Total antioxidant capacity was measured as the ferric reducing ability of plasma (FRAP). In this method ferric-tripyridyltriazine (Fe^3+^-TPTZ) complex is reduced to the ferrous (Fe^2+^) form at low pH by the plasma antioxidants. Reduced Fe^2+^-TPTZ takes on an intense blue color, with the peak absorbance at 593 nm [11]. All spectrophotometric measurements for the determination of glutathione peroxidase and glutathione reductase activity and the concentration of reduced glutathione were performed on the MaxMat PL analyzer (Maxmat SA, Montpellier, France).

*Statistics.* Normality of distribution of obtained variables was checked using the Shapiro Wilk test. Data are expressed as mean ± SD, median and interquartile range, or percentage of frequency, as appropriate. Relationships between paired parameters (continuous variables) were analyzed by Pearson product moment correlation coefficient. Variables with positively skewed distribution were log transformed before the correlation analysis. Differences between groups were assessed by *t*-test for variables with positively skewed distribution and Mann-Whitney *U* test for nonparametric variables. *p* values less than 0.05 were taken as statistically significant. All analyses were performed using a standard statistical package Statistica 9.0 (StatSoft Inc.) and Microsoft Office Excel 2007 spreadsheet (Microsoft Corporation).

## 3. Results

The characteristics of the studied groups are shown in Table 1. The studied group was characterized by hyperglycemia and elevated glycated hemoglobin. Mean values of lipid profile parameters did not fit treatment goals recommended by diabetes associations including the Polish Diabetes Society [12]. Mean BMI value was elevated and 55% of patients were obese (BMI > 30 kg/m^2^).

The results of antioxidant defense markers measurements in patients with diabetes and controls are shown in Table 2.

In subjects with type 2 diabetes significantly lower levels of glutathione peroxidase and higher levels of glutathione reductase both in plasma and hemolysate were found. The trend towards decreased hemolysate reduced glutathione concentrations in the group with diabetes compared with controls (0.87 (0.46–2.23) *μ*mol/L versus 0.92 (0.56–1.20) *μ*mol/L) was observed.

**Figure 1:**
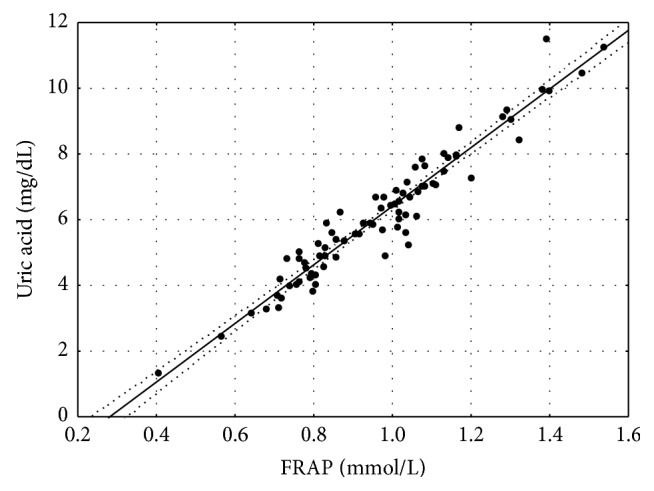
Correlation between FRAP and uric acid in people with type 2 diabetes (*r* = 0.963; *p* ≤ 0.001).

**Figure 2:**
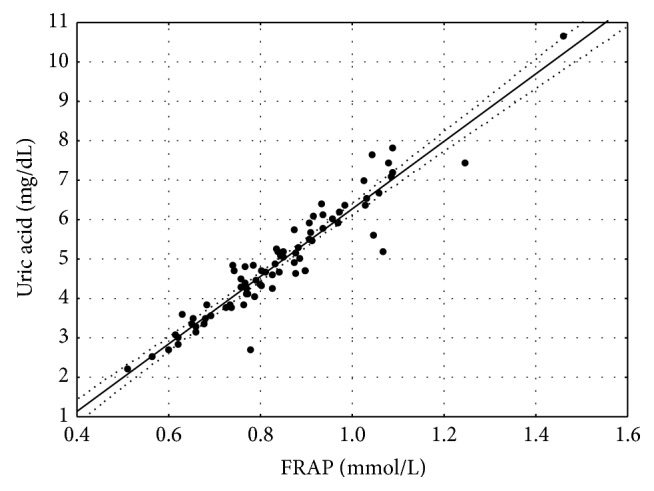
Correlation between FRAP and uric acid levels in the control group (*r* = 0.948; *p* ≤ 0.001).

Total antioxidant capacity of plasma assessed by the FRAP, serum uric acid, and GGT, also involved in antioxidant defense, were significantly higher in patients with diabetes (Table 2). Plasma uric acid concentration was significantly correlated with FRAP both in patients and in the control group (Figures 1 and 2).

Fasting serum glucose correlated with glutathione peroxidase in plasma (*r* = −0.2816, *p* < 0.001), glutathione reductase in plasma and hemolysate (*r* = −0.2816, *p* < 0.001; *r* = 0.6010, *p* < 0.001), FRAP (*r* = 0.3816, *p* < 0.001), and GGT (*r* = 0.4565, *p* < 0.001). Moreover correlation between GGT and HbA1c (*r* = 0.4179, *p* < 0.001) was found in diabetic patients.

In patients with chronic diabetes complications (at least one) no significant differences in antioxidant defense markers as compared to those without complications were found (Table 3). However, for uric acid correlation with albuminuria was found (*r* = 0.46, *p* = 0.02).

The relationship between metabolic syndrome features present in studied patients and antioxidant defense markers was also evaluated. No significant differences in these markers levels between obese (BMI > 30 kg/m^2^) and nonobese patients with diabetes were found except for the FRAP and uric acid concentrations significantly higher in obese (FRAP: 1.03 ± 0.2 mmol/L versus 0.89 ± 0.18 mmol/L; *p* = 0.002; UA: 6.7 ± 2.0 *μ*mol/L versus 5.4 ± 1.7 *μ*mol/L, *p* = 0.003) and significantly correlated with BMI (FRAP: *r* = 0.593, *p* < 0.001; UA: *r* = 0.415, *p* < 0.001). Hypertensive patients with diabetes had significantly higher glutathione reductase activity in hemolysate as compared to those without hypertension (38.6 U/L versus 32.7 U/L, *p* = 0.03).

## 4. Discussion

Diabetes is a state of increased oxidative stress, which is one of the mechanisms leading to development of chronic diabetes complications [1]. While it is believed that diabetes is associated with an increased production of ROS, the reports of the antioxidant defense in diabetes are contradictory [5, 6]. In this study markers of antioxidant defense in patients with type 2 diabetes were evaluated. The study included a heterogeneous group of patients with type 2 diabetes—with and without chronic complications, and a part of patients had obesity, hypertension, and other features of the metabolic syndrome. Results obtained in this study showed that patients with diabetes had significantly higher antioxidant defense activity reflected by FRAP, glutathione reductase and gamma-glutamyltransferase activity, and uric acid levels. Glutathione peroxidase activity measurements in plasma and hemolysate yielded significantly lower results in the patients group (Table 2). Similarly, an inverse relationship between glutathione peroxidase and glutathione reductase in patients with type 2 diabetes and the control group was observed by Kumawat et al. [13]. In another study decreased activity of both glutathione peroxidase and glutathione reductase in type 2 diabetes was found [14].

The low activity of GPx could be directly explained by the low content of GSH found in patients with type 2 diabetes, since GSH is a substrate and cofactor of GPx. Enzyme inactivation could also contribute to low GPx activity. GPx is a relatively stable enzyme, but it may be inactivated under conditions of severe oxidative stress. Inactivation of this enzyme may occur through glycation governed by prevailing glucose concentration [15]. Increased activity of GR may be a compensatory response to oxidative stress. Changes in glutathione peroxidase and glutathione reductase activity found in this study can be considered an adaptation of antioxidant defense against increased production of ROS. However, this adaptation seems to be ineffective. Reduced GSH level in red blood cells reflects generalized decrease in intracellular content of this compound. The obtained results showed that patients with type 2 diabetes had lower GSH content in erythrocytes than observed in the control group. However, this difference was not statistically significant. The trend found in our results is consistent with the report of Aaseth and colleagues, who found reduced levels of glutathione in erythrocytes of subjects with obesity and poorly controlled type 2 diabetes [16]. Similarly, decrease in intracellular glutathione levels in patients with type 2 diabetes was reported by Livingstone in his recent review [17]. These results indicate that patients with type 2 diabetes have lower concentration of intracellular GSH, which increases the susceptibility of cells to the damaging effects of ROS.

There were no significant differences in oxidative defense markers between patients with and without chronic diabetes complications.

Plasma glutathione reductase, plasma glutathione peroxidase, FRAP, and GGT were associated with fasting glucose concentration, which indicated the association of higher level of antioxidant defense with hyperglycemia and subsequent ROS production. This activation of antioxidant defense seems to be associated with short-term glycemia fluctuations, because no relationship between antioxidant defense markers and HbA1c was found.

Metabolic syndrome includes risk factors for development of type 2 diabetes—hypertension and atherogenic dyslipidemia (elevated triglycerides and decreased HDL cholesterol concentrations). In obese patients serum uric acid levels were significantly higher as compared with those without obesity. We also observed significant positive correlation between BMI and serum uric acid concentrations and negative with plasma glutathione peroxidase activity. Decreased glutathione peroxidase activity associated with increased BMI is reported as a feature of oxidative stress in obese individuals [18].

In the whole studied group significant correlation between uric acid concentration and plasma total antioxidant capacity both in patients (*r* = 0.963) and in the control group (*r* = 0.948) was found. This observation confirms the well-known relationship between the level of antioxidant capacity measured as FRAP and plasma uric acid [11]. Moreover, serum uric acid concentrations correlated significantly with HDL cholesterol (negatively) and triglycerides (positively) levels. Similarly, increased levels of uric acid were reported by Chen et al. to be associated with abnormal levels of HDL [19]. Increase in uric acid concentrations is a well-known abnormality observed in metabolic syndrome. The underlying mechanisms are not fully explained. Recently it was demonstrated that leptin may influence hyperuricemia associated with obesity. Uric acid is considered a risk factor for diabetes complications. The study by Hovind et al. showed that uric acid is an independent risk factor for the development of diabetic nephropathy in type 1 diabetes. Moreover, in this study correlation between serum uric acid and albuminuria was found [20]. Similar results were reported for patients with type 2 diabetes [21]. Significant correlation between serum uric acid and albuminuria was demonstrated in our study but the limitation is the small number of albuminuric patients studied. Regardless of these relationships uric acid remains an important component of antioxidant defense.

Another enzyme involved in glutathione metabolism and the overall antioxidant defense system is GGT responsible for glutathione uptake from the extracellular fluid into the cells to maintain its constant level. Recently the relationship between plasma GGT activity and obesity with the risk for type 2 diabetes was reported [22]. In our study the mean GGT activity was significantly higher in diabetes individuals but without significant correlation with BMI.

Monitoring antioxidant defenses may also be important in clinical practice. Since the increased antioxidant defense may alleviate oxidative stress, supplementation with antioxidants has been considered an attractive potential therapy. This approach has been evaluated in numerous clinical trials assessing the effect of antioxidants supplementation on the risk for diabetes, glycemic control, and the development of chronic complications. However, the results of these studies are contradictory and do not allow us to draw consistent conclusion [23–29]. Nevertheless, our results indicating insufficient activation of antioxidant defense in type 2 diabetes may argue for the usefulness of such supplementation.

In summary, results obtained in this study show that some components of antioxidant defense such as glutathione reductase, uric acid, and GGT are increased in type 2 diabetes. However, this system seems to be ineffective as reflected by the trend toward decreased reduced glutathione content in erythrocytes. Although increased, the whole system of antioxidant defense cannot compensate for an enhanced production of ROS in diabetes, which results in oxidative stress. These data may indicate the usefulness of therapies enhancing antioxidant defense.

## Figures and Tables

**Table 1:** Demographic and biochemical characteristics of studied patients with type 2 diabetes and controls.

Parameter	Type 2 diabetes *n* = 80	Control group *n* = 79
Age [years]	59.5 ± 11.6	55.2 ± 11
Gender		
Women	40 [50]	40 [51]
Men	40 [50]	39 [49]
Diabetes duration [years]	8.5 (4.0–12)	—
BMI	31.54 ± 5.73	29.26 ± 8.43
Hypertension	77 [88.7]	—
Obesity (BMI > 30 kg/m^2^)	44 [55]	32 [41]
No complications	38 [47.5%]	—
Complications	42 [52.5%]	—
Glucose [mmol/L]	6.75 (5.25–9.35)	4.4 (4.0–4.9)
HbA1c [%] [mmol/mol]	8.5 (7.25–10.0) 69 (56–86)	—
Creatinine [*μ*mol/L]	66.8 (58.2–82.0)	—
e-GFR (MDRD) [mL/min/1.73 m^2^]	94.8 (75–116.4)	—
TCH [mmol/L]	4.38 (3.82–5.27)	5.06 (4.09–5.9)
LDL [mmol/L]	2.21 (1.81–3.15)	2.94 (2.22–3.75)
HDL [mmol/L]	0.82 (0.71–1.00)	1.29 (1.06–1.24)
TG [mmol/L]	2.55 (1.74–3.75)	1.47 (1.08–1.87)
Albuminuria [mg/L]	2.51 (0.0–8.03)	—

Data are expressed as mean ± SD, median and interquartile range, or percentage of frequency [%], as appropriate.

**Table 2:** Comparison of antioxidant defense markers in patients with type 2 diabetes and controls.

Parameter	Type 2 diabetes	Control group	*p*
FRAP [mmol/L]	0.97 ± 0.21	0.84 ± 0.16	**<0.001**
GPx_plasma_ [U/L]	235.7 (93.2–359.9)	644.5 (466.0–798.8)	**<0.001**
GPx_hemolysate_ [U/gHb]	43.8 ± 21.5	55.4 ± 25.9	**0.002**
GR_plasma_ [U/L]	74.5 ± 21.3	43.7 ± 13.5	**<0.001**
GR_hemolysate_ [U/gHb]	33.0 (26.9–38.5)	22.62 (16.2–29.1)	**<0.001**
GSH [*μ*mol/L]	0.87 (0.46–2.23)	0.92 (0.56–1.20)	0.117
Uric acid [*μ*mol/L]	364.3 ± 117.9	293.5 ± 85.7	**<0.001**
GGT [U/L]	23.5 (11.5–37.5)	10.5 (7.0–17.0)	**<0.001**
CRP [mg/dL]	1.95 (0.85–5.72)	0.87 (0.46–2.23)	**<0.001**

Data are expressed as mean ± SD and median and interquartile range.

**Table 3:** Comparison of selected clinical and biochemical parameters in patients with and without chronic diabetes complications.

Parameter	Complications (*N* = 42)	No complications (*N* = 38)	*p*
Age [years]	60.9 ± 9.0	57.9 ± 13.9	0.009
BMI	30.92 ± 5.24	32.22 ± 6.22	0.315
Diabetes duration [years]	10 (7–16)	5 (1–9)	**<0.001**
Mean daily glucose [mmol/L]	6.3 ± 1.0	6.3 ± 1.1	0.651
HbA1c [mmol/mol]	69.4 (58.5–74.9)	77.1 (51.9–92.4)	0.372
[%]	8.5 (7.5–9.0)	9.2 (6.9–10.6)	
Creatinine [*μ*mol/L]	66.4 (59.4–76.0)	67.8 (56.1–83.0)	0.891
FRAP [mmol/L]	1.00 ± 0.22	0.94 ± 0.20	0.512
GPx_plasma_ [U/L]	234.5 (89.2–60.5)	236.9 (98.4–59.3)	0.950
GPx_hemolysate_ [U/gHg]	43.1 ± 22.0	44.6 ± 21.1	0.819
GR_plasma_ [U/L]	73.1 ± 18.2	75.5 ± 24.5	0.063
GR_hemolysate _[U/gHg]	33.1 (27.1–38.6)	32.8 (26.7–37.9)	0.969
Glutatione [*μ*mol/L]	0.88 (0.53–1.18)	0.94 (0.71–1.21)	0.402
Uric acid [*μ*mol/L]	381.0 ± 131.0	345.2 ± 71.4	0.170
GGT [U/L]	22.5 (12–38)	25.5 (10.0–34.0)	0.711
CRP [mg/L]	1.7 (0.5–4.4)	2.7 (1.1–10.0)	0.117

Data are expressed as mean ± SD, median and interquartile range, or percentage of frequency [%], as appropriate.

## Abbreviations

FRAP: Ferric reducing ability of plasma
GP: Glutathione peroxidase
GR: Glutathione reductase
GGT: *γ*-Glutamyltransferase
CRP: C-reactive protein
HBA1c: Glycated hemoglobin
ROS: Reactive oxygen species
O_2_^−^: Superoxide anion radical
AGE: Advanced glycation end products
NADPH: Reduced nicotinamide adenine dinucleotide phosphate
NADP^+^: Oxidized nicotinamide adenine dinucleotide phosphate
GSH: Reduced glutathione
GSSG: Glutathione disulfide (oxidized glutathione)
BMI: Body mass index
UA: Uric acid
HDL: High-density lipoprotein
LDL: Low-density lipoprotein
TG: Triglyceride
TCH: Total cholesterol
NF-*κ*B: Nuclear factor kappa B
GAPDH: Glyceraldehydes -3-phosphate dehydrogenase
G-6PDH: Glucose-6-phosphate dehydrogenase
IGT: Impaired glucose tolerance.
